# Time spent lying, sitting, and upright during hospitalization after stroke: a prospective observation study

**DOI:** 10.1186/s12883-018-1134-0

**Published:** 2018-09-04

**Authors:** Ole Petter Norvang, Anne Hokstad, Kristin Taraldsen, Xiangchun Tan, Stian Lydersen, Bent Indredavik, Torunn Askim

**Affiliations:** 1 Department of Neuromedicine and Movement Science, Faculty of Medicine and health science, NTNU - Norwegian University of Science and Technology, Trondheim, Norway; 2 Clinical Services, Department of Physiotherapy, St Olavs Hospital, Trondheim University Hospital, NO-7006 Trondheim, Norway; 3 Clinic of Medicine, Department of Stroke, St. Olavs Hospital, Trondheim University Hospital, Trondheim, Norway; 4 Regional Centre for Child and Youth Mental Health and Child Care, Department of Mental Health, Faculty of Medicine, NTNU - Norwegian University of Science and Technology, Trondheim, Norway

**Keywords:** Stroke, Early rehabilitation, Accelerometer, Physical activity

## Abstract

**Background:**

Early mobilization has been an important part of acute stroke unit treatment. However, early and intense mobilization within the first 24 h post stroke may cause an unfavorable outcome. Recently, objective measurements using body-worn sensors have been applied, enabling continuous monitoring of physical activity in the hospital setting. This study aimed to use body-worn sensors to quantify the amount of physical activity and how activity levels changed over time during hospitalization in patients with acute stroke. We also wanted to investigate which factors were associated with upright and sitting activity.

**Methods:**

This was a prospective study including patients admitted to hospital within seven days after onset of stroke. Physical activity was measured by two sensors (ActivPALs from PAL Technologies Ltd., Glasgow, UK), one attached on sternum and one on the thigh of the unaffected side, monitoring continuously from inclusion until discharge. Data were processed in Matlab R 2015B and provided information about daily time in lying, sitting, and upright positions, and daily average duration of sitting and upright bouts. A linear mixed model was used to analyze changes over time.

**Results:**

58 patients were included (31 women, mean (SD) age; 75.1 (12.0)). Patients were hospitalized for 12.1 (7.6) days and had a mean score on the National Institute of Health Stroke Scale of 6.2 (5.5) points. Time spent sitting and time spent upright increased per day during hospitalization by 22.10 min (95% Confidence interval (CI): 14.96, 29.24) and 3.75 min (95% CI: 1.70, 5.80) respectively. Increased time upright was associated with improved Modified Rankin Scale scores (− 38.09 min, 95% CI: -61.88, − 14.29) and higher Short Physical Performance Battery scores (6.97 min, 95% CI: 1.99, 11.95), while prolonged bouts of sitting were associated with more severe stroke (4.50 min, 95% CI: 0.80, 8.19), and older age (1.72 min, 95% CI: 0.20, 3.26).

**Conclusions:**

Patients increased their daily time spent sitting and upright during the initial hospital stay after stroke. Prolonged bouts of sitting were associated with older age and more severe strokes. Hence future research should investigate the benefit of interventions aimed at breaking up sitting time after stroke.

## Background

One of the core elements of care for dedicated comprehensive stroke units is acute medical treatment combined with early rehabilitation [1–3]. Early rehabilitation with mobilization out of bed during hospitalization has shown to be associated with better functional outcomes for patients after stroke [4–8]. This may reduce the loss of muscle mass, increase muscle strength [6, 9], avoid complications [3, 4, 10], exploit the plasticity of the brain [11], improve neurological functioning [12], and improve gait function [13]. Although time spent out of bed early after stroke varies significantly between hospitals [14–16], the amount of physical activity is generally low during hospital stays [17, 18].

Optimal timing and intensity of early mobilization is, however, not clear, as previous studies have shown an association between mobilization within the first 24 h and a negative trend towards increased dependency [12, 19] and increased mortality [20]. On the other hand, short and frequent mobilizations have been associated with improved outcome [21].

Most studies of physical activity during hospitalization have been single-day observational studies during daytime hours. Recently, objective measurements using body-worn single sensor activity monitors have been applied, enabling continuous monitoring of physical activity over time [22, 23]. So far, only a few studies have used activity monitors to distinguish between different activities in the acute phase for stroke patients [24]. In one of these studies, Strømmen et al. (2014) found significantly increased activity in the lower extremities for hospitalized stroke patients from the first day until discharge [25]. The increased activity was significantly associated with lower severity of stroke, whereas decreased activity was found with increasing age [25]. However, the study did not discriminate between times spent in different positions, which is crucial when measuring physical activity in the acute phase after stroke. Applying two single sensors from the ActivPAL sensor system, one on the chest and one on the thigh, makes it possible to quantify the amount of time spent in different positions. This method has previously been proved valid for stroke patients [26].

The main aim of this study was to describe the amount of time spent in lying, sitting and upright (standing or walking) positions early after stroke and how these activity levels changed during the hospital stay, both regarding total time per day and duration for each mobilization. Another aim was to examine which factors were associated with total time and bouts of sitting and upright activity. Our primary hypothesis was that patients would gradually increase time spent in an upright position, and thereby increase the duration of each upright bout.

## Method

### Design

This study used a prospective observation design, measuring physical activity continuously with two activity monitors for three to seven consecutive days during hospitalization.

### Study setting

All patients were treated in an evidence-based comprehensive stroke unit that emphasizes a multidisciplinary approach and early rehabilitation. The treatment focused on independence in daily life. All patients, regardless of participation, received routine medical treatment, including rehabilitation, in accordance with Norwegian guidelines for treatment and rehabilitation after stroke [27].

### Study population

Patients were recruited during three time periods, October–December 2013, May–December 2014, and March–August 2016. Patients admitted to Trondheim University Hospital, Norway, with diagnosed first ever or recurrent acute ischemic or hemorrhagic stroke were eligible for inclusion if the onset of stroke had been within seven days, they spoke fluent Norwegian, and if they gave written consent. In line with the Norwegian regulations for informed consent, patients who were unable to consent were included if their next of kin not opposed participation. Patients with terminal illness, other health conditions severely affecting their ability to walk, or expected discharge within three days after inclusion were excluded.

### Measurements

The ActivPAL Professional sensor system (from PAL Technologies Ltd) consists of a three-axis accelerometer that collects continuous data with a sampling frequency of 10 Hz, and with a battery capacity of up to 14 days. The primary outcome for this study was whether time spent lying, sitting or upright changed during the hospital stay. It has previously been shown that this activity monitor provides valid data for time spent lying, sitting, standing and walking [28, 29] and that placement on the thigh and sternum provides good validity for postures and transitions compared to video observations [26]. The 24-h period was measured from the time the activity monitors were attached. Duration of sitting bouts was estimated according to the following ratio: time spent in sitting/number of transitions from lying to sitting, while duration of upright bouts was estimated correspondingly. A time threshold for transitions was set at 1.5 s to eliminate unreliable event records.

The Short Physical Performance Battery (SPPB) (ranging from 0 to 12, where 12 is the best score) was used to assess physical function. The test consists of three different mobility tasks, and has been found to be both valid and reliable for assessing physical function amongst elderly people [30]. Gait speed was calculated based on the walking task of the SPPB. An experienced nurse or physiotherapist performed the test.

Global function was defined using the Modified Rankin Scale (mRS) (ranging from 0 to 6, where 0 is normal function and 6 denotes death) [31] at admission and after the first day in hospital. The mRS measures independence in activities of daily living.

The National Institutes of Health Stroke Scale (NIHSS) was used to measure severity of the stroke. The scale is widely used and has proved both valid and reliable [32].

Both mRS and NIHSS were scored by an experienced clinician within the first day after the patient’s admission and recorded together with age, gender, number of days from first symptom to admission date, and number of days at the hospital, which were collected from the medical record.

### Procedure

All patients accepting participation had two ActivPAL activity monitors attached, one at the sternum and one at the unaffected thigh, to distinguish between lying, sitting and upright position. If neither of the lower extremities were affected by the stroke, the activity monitor was attached to the right thigh. Patients were instructed to follow the standard rehabilitation routines without paying attention to the equipment. Both activity monitors were removed 14 days post stroke unless the patient had been discharged earlier. Data from the activity monitors were transferred via a USB docking station, and processed in Matlab R2015 B. The valid body and leg data had an individual start and stop time defined and used to measure a count for a full 24-h day, and the activity was coded in reference to the body position. To distinguish the different positions, data from the two sensors were synchronized and recoded according to body position (lying, sitting, and upright).

State durations were calculated by accumulating time intervals for lying, sitting, and upright and state transitions between these positions per 24-h measurement. Time upright was calculated by merging time standing and time walking. A validation procedure was performed to control for possible time drifting of the sensors.

### Statistical analysis

Demographic data were reported as mean values and standard deviation (SD) for all patients, and for three subgroups categorized by time from onset of symptoms to inclusion. Baseline characteristics were compared for those included within the first two days after stroke, those included 3 to 4 days after stroke and those included 5 to 7 days after stroke. This was done using a one-way ANOVA or Kruskal-Wallis test, depending on normality of residuals, which was judged by visual inspection of Q-Q plots. To assess changes in activity levels, we used linear mixed models (LMM) with time sitting, time upright, duration of sitting bouts and duration of upright bouts, respectively, as dependent variables, patients as random factor, and days since stroke as covariate. Next, we included the following covariates, one at a time: NIHSS, age, gender, mRS pre-stroke, mRS one day post stroke, and SPPB. Because the time spent lying, sitting and upright totaled 24 h, only time spent sitting and upright were used in the mixed model analysis. Two-sided *p*-values less than 0.05 were considered statistically significant, and 95% confidence intervals (CI) are reported where relevant. Statistical analyses were done in SPSS 23.

## Results

**Fig. 1:**
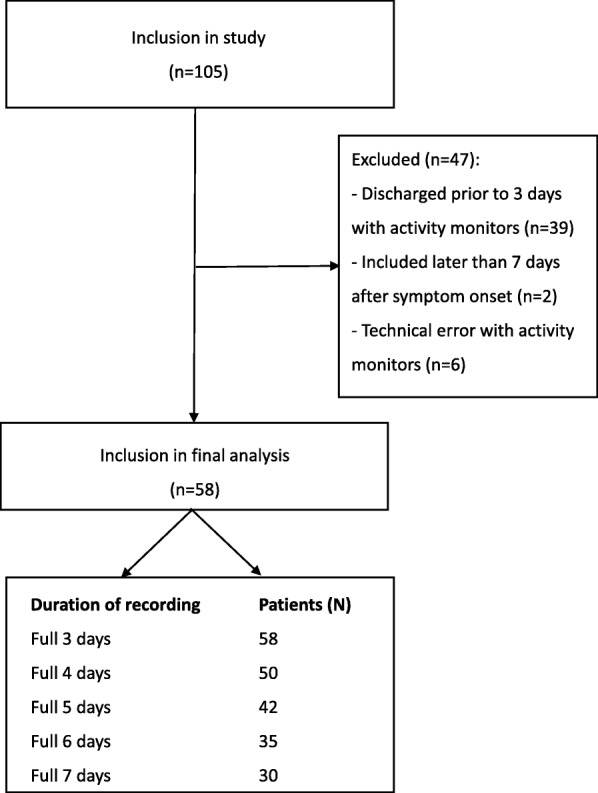
Flow chart

The flow of patients is shown in Fig. 1. Of the 105 patients who met the inclusion criteria, 47 were excluded, mainly because of early discharge.

Fifty-eight patients (31 female) were included in the analysis. Patient characteristics are presented in Table 1. Mean (SD) time from onset of symptoms to inclusion was 2.6 (1.7) days, and patients wore the activity monitors for 5.8 (1.5) days. The NIHSS score was 6.2 (5.0) points. Those included 4–7 days after symptom onset showed significantly lower scores on NIHSS as compared to those included within the first 24 h (*p* = 0.002). No other significant differences were found for the baseline variables.

**Table 1:** Demographic data for included patients (*N* = 58)

	Included 0–2 days from symptoms (*n* = 19)	Included 3–4 days from symptoms (*n* = 24)	Included 5–7 days from symptoms (*n* = 15)	Total (*n* = 58)
Female gender, *n* (%)	11 (57.9)	11 (45.8)	9 (60.0)	31 (53.5)
Age (years)	72.9 (10.0)	76.6 (13.1)	75.3 (12.1)	75.1 (12.0)
Monitoring days	5.8 (1.6)	5.8 (1.6)	6.0 (1.3)	5.8 (1.5)
Days hospitalized	10.6 (4.6)	10.0 (4.6)	12.6 (5.5)	12.1 (7.6)
Days from stroke to inclusion	0.9 (0.3)	2.4 (0.5)	5.0 (1.3)	2.6 (1.7)
NIHSS when admitted	8.4 (4.4)	8.4 (4.4)	4.6 (6.8)	6.2 (5.0)
Median (IQR)	7.0 (6.0–13.0)	4.0 (3.0–7.0)	3.0 (2.0–5.0)	5.0 (3.0–8.0)
Stroke severity groups, *n* (%)				
Mild (NIHSS < 8)	13 (68.4)	19 (79.2)	14 (93.3)	46 (79.1)
Moderate (NIHSS 8–16)	6 (31.6)	5 (20.8)	0 (0.0)	11 (19.0)
Severe (NIHSS > 16)	0 (0.0)	0 (0.0)	1 (6.7)	1 (1.7)
mRS prior to stroke	1.6 (1.1)	1.6 (1.1)	1.2 (0.9)	1.7 (1.2)
Median (IQR)	1.0 (1.0–2.0)	2.0 (2.0–3.0)	1.0 (1.0–2.0)	2.0 (1.0–2.3)
mRS when admitted	4.1 (0.5)	4.1 (0.5)	4.1 (0.7)	4.1 (0.7)
Median (IQR)	4.0 (4.0–4.0)	4.0 (4.0–5.0)	4.0 (4.0–4.0)	4.0 (4.0–4.3)
Gait speed (m/s)	1.7 (1.6)	1.5 (0.9)	1.2 (1.2)	1.5 (1.2)
SPPB	5.1 (3.8)	5.1 (3.8)	3.9 (3.5)	4.4 (3.5)
Median (IQR)	5.0 (1.0–7.0)	3.5 (2.0–7.0)	4.0 (0.0–7.0)	4.0 (1.0–7.0)

Mean (SD) unless otherwise stated

*NIHSS* National Institute of Health Stroke Scale, *mRS* modified Rankin Scale, *SPPB* Short Physical Performance Battery

**Fig. 2:**
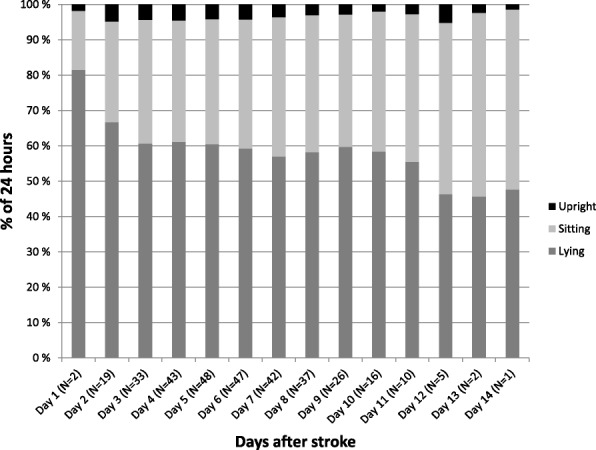
Percentage of time spent in different positions within every 24 h

Figure 2 illustrates the combinations of activities for each day, showing an increase in time spent out of bed during the hospital stay. Time spent upright remained low throughout the observation period.

Results from the LMM analysis (Table 2) show a daily increase in time spent sitting of 22.10 min (*p* < 0.001) and 3.75 min upright (*p* < 0.001). Adjusted for time, we found that decreasing time spent upright was associated with increasing dependency, measured by mRS the first day after stroke (*p* = 0.002). An association was also found between time spent upright and physical function showing an increase of 6.97 min upright for every point increase on SPPB (*p* = 0.007). Neither severity of stroke nor age significantly influenced changes in overall physical activity.

**Table 2:** Linear mixed model regression with activity level as dependent variable

	Time sitting (minutes)			Time upright (minutes)			Duration of bouts in sitting (minutes)			Duration of bouts in upright (minutes)		
Covariate	Coefficient	95% CI	*p*-value	Coefficient	95% CI	*p*-value	Coefficient	95% CI	*p*-value	Coefficient	95% CI	*p*-value
Time stroke to inclusion (days)^a^	22.10	14.96, 29.24	< 0.001	3.75	1.70, 5.80	< 0.001	4.32	−0.25, 8.66	0.051	0.14	−0.08, 0.36	0.212
NIHSS	0.19	−10.53, 10.91	0.972	−2.34	−5.97, 1.28	0.201	4.50	0.80, 8.19	0.018	−0.08	−0.35, 0.19	0.547
Age (years)^b^	3.09	−1.26, 7.44	0.160	−0.47	−1.99, 1.04	0.534	1.72	0.20, 3.26	0.028	−0.08	−0.19, 0.03	0.133
Female gender^b^	− 35.23	− 141.08, 70.60	0.508	−12.55	−48.93, 23.83	0.492	21.10	−16.95, 59.14	0.271	−0.90	−3.52, 1.71	0.493
mRS_pre strole^b^	− 2.48	−47.02, 42.06	0.912	1.72	−13.60, 17.03	0.823	9.52	−6.39, 25.43	0.236	0.09	−1.00, 1.19	0.867
mRS_post stroke^b^	− 66.98	−140.22, 6.27	0.072	−38.09	−61.88, − 14.29	0.002	6.83	− 20.29, 33.96	0.616	−0.73	−2.57, 1.11	0.432
SPPB	12.21	−2.92, 27.33	0.111	6.97	1.99, 11.95	0.007	−3.23	−8.78, 2.33	0.250	0.18	−0.20, 0.55	0.358

^a^Unadjusted

^b^Adjusted for days since stroke

Although not significant, LMM analysis indicated a daily increased duration of 4.32 min for each sitting bout (*p* = 0.051). Adjusted for time, this increase in time was associated with both a higher NIHSS score (*p* = 0.018) and increasing age (*p* = 0.028).

## Discussion

In this study, we found that stroke patients increased their time spent sitting and upright during the initial hospital stay, with a corresponding decrease in time spent in a lying position. The duration of sitting bouts also increased over time, while the duration of upright bouts remained constant. Furthermore, overall increased time spent upright was associated with increasing independency (mRS) and improved physical function (SPPB), while prolonged sitting bouts were associated with higher age and more severe stroke.

Despite a statistically significant increase in time spent upright during the hospital stay, we may ask whether an estimated 3.75 min’s more upright activity per day was a clinically significant change. This means that time spent upright will increase by almost 40 min during a 10-day hospital stay. A previous study has shown that every 5-min increase in time spent in bed was associated with a 4% deterioration on the mRS score three months later [6]. Hence, the daily increase in time spent upright could potentially have a great impact on functional recovery over time.

On the other hand, we found no association between stroke severity and time spent out of bed. This finding might be explained by the fact that activity measures during hospitalization also mirror the mobilization routines in the wards [33], showing that patients are mobilized out of bed as soon as they are medically approved, independent of the patients’ ability to mobilize themselves.

The duration of each sitting bout also increased by more than 4 min per day during the hospital stay. More surprisingly, the increased duration of sitting bouts was associated with higher age and more severe stroke, while no associations were found for bouts in upright activity. These findings might be explained by the ability of less affected and younger patients to change position more often by themselves. This is interesting because it adds further knowledge to the ongoing debate regarding the intensity of early mobilization. Also, clinicians should be aware that patients with the most severe strokes who are not able to move independently are at increased risk of sedentary behavior, which might be harmful, thereby increasing the risk of poor outcome [6].

Our results show that stroke patients on average spend a significant proportion of each 24 h in a lying position (Fig. 2). Considering the 24-h monitoring, patients would normally spend at least 30% of the time sleeping. Still, patients spent almost 30% of the remaining time in bed. This corresponds with the high amount of time spent lying previously reported early after stroke [14, 17, 25]. One factor that could influence the low overall activity amongst our participants is the exclusion of those expected to be discharged within three days after inclusion, as these patients are likely to be more active. However, as we aimed to study change in physical activity over time, patients with short monitoring time had to be excluded.

The major strength of the present study was the prospective study design with continuous monitoring of activity, from two activity monitors, over 24 h for several days during the hospital stay. This allowed us to discriminate between lying, sitting and upright positions, which is of major importance in the acute phase after stroke. The use of such a protocol has been validated in the stroke population earlier, showing a high accuracy compared to video observation [26]. In contrast to observation of activity by, for example, behavioral mapping during daytime hours, usually for 9–10 h [7, 14, 17], continuous monitoring will account for out-of-bed time during 24 h. It is also a strength that all included patients received evidence-based treatment in a comprehensive stroke unit combining acute medical treatment and early rehabilitation. Therefore, we know that our patients were mobilized according to recommendations in the national and international guidelines [27, 34], increasing the validity of the study.

A limitation of this study was the wide window for inclusion, ranging from one to seven days post stroke. This criterion was chosen for practical reasons, as recruitment was delayed during weekends and because the diagnosis was delayed in some patients (if the MR images were inconclusive or if patients were not admitted to hospital immediately after the first stroke symptoms). Nevertheless, apart from a significant difference in NIHSS between early and late admission to hospital, indicating that patients with the most severe symptoms are admitted to hospital earlier, we found no significant differences in the demographic data at baseline. However, the variation in time from onset of stroke to inclusion is accounted for in the LMM analysis. Our prolonged recruitment period (2013–2016), with three different time periods may also have influenced our results. The same routines were, however, applied for all stroke patients in the acute stroke treatment in the time periods. Therefore, all included patients, regardless of when they were recruited, should have received treatment focused on early rehabilitation. Another limitation is the lack of possibility to draw a causal relationship between the clinical parameters and the activity categories. Our secondary analysis indicate that the clinical parameters were influencing the activity levels. However, it is also possible to reverse this relationship, and to argue that the activity levels might influence the clinical parameters. Hence, it is important to have this possible bilateral relationship in mind when interpreting the results. Finally, the accelerometers used in this project do not discriminate between active and passive mobilization, and based on our inclusion criteria, mobilization may have been conducted with or without support from another person. This could influence both length and frequency of mobilization, as an active mobilization is naturally more tiring for the patient. However, in a comprehensive stroke unit, hospital staff are trained to activate stroke patients as soon as medically accepted, to encourage active rehabilitation. This means that even though some patients receive more support than others, all patients should be challenged according to their functional level.

### Clinical implications

Our study supports previous findings showing that stroke patients spend most of their time in a lying or sitting position [7, 17, 25, 28], during hospitalization. The association between increased time spent upright and improved function indicates a need to pay specific attention to patients who depend on support to be able to transfer to an upright position to ensure frequent and short periods in upright activity throughout their hospital stay as recommended [21]. As older and more severely affected stroke patients may not be able to change position as often as independent patients, hospital staff also should pay extra attention to these patients when mobilizing them to a sitting position, to reduce sedentary time and avoid complications.

## Conclusion

This study showed that time spent in sitting and upright positions increased throughout the hospital stay in patients admitted to hospital following a stroke. We found that these changes were associated with improved physical function and a higher degree of independence. There was also an increased duration of bouts spent sitting during hospitalization, with longer bouts associated with increasing age and more severe strokes. Future research should focus on defining the optimal dose of activity in the acute phase in order to improve function in the long term after stroke.

## Abbreviations

CI: Confidence Interval
IQR: Inter Quartile Range
LMM: Linear Mixed Model
MRS: Modified Rankin Scale
NIHSS: National Institute of Health Stroke Scale
SD: Standard Deviation
SPPB: Short Physical Performance Battery
