# Correction to: Time spent lying, sitting, and upright during hospitalization after stroke: a prospective observation study

**DOI:** 10.1186/s12883-018-1226-x

**Published:** 2019-01-11

**Authors:** Ole Petter Norvang, Anne Hokstad, Kristin Taraldsen, Xiangchun Tan, Stian Lydersen, Bent Indredavik, Torunn Askim

**Affiliations:** 1 Department of Neuromedicine and Movement Science, Faculty of Medicine and health science, NTNU - Norwegian University of Science and Technology, Trondheim, Norway; 2 Clinical Services, Department of Physiotherapy, St Olavs Hospital, Trondheim University Hospital, NO-7006 Trondheim, Norway; 3 Clinic of Medicine, Department of Stroke, St. Olavs Hospital, Trondheim University Hospital, Trondheim, Norway; 4 Regional Centre for Child and Youth Mental Health and Child Care, Department of Mental Health, Faculty of Medicine, NTNU - Norwegian University of Science and Technology, Trondheim, Norway

**Correction to: BMC Neurol (2018) 18:138**

**https://doi.org/10.1186/s12883-018-1134-0**

The original version of this article [1] unfortunately contained errors. The authors wish to correct the Gait speed (m/s) values in Table 1 and add a corresponding table footnote. Below is the correct version of the table.

**Table 1:** Demographic data for included patients (*N* = 58)

	Included 0–2 days from symptoms (*n* = 19)	Included 3–4 days from symptoms (*n* = 24)	Included 5–7 days from symptoms (*n* = 15)	Total (*n* = 58)
Female gender, *n* (%)	11 (57.9)	11 (45.8)	9 (60.0)	31 (53.5)
Age (years)	72.9 (10.0)	76.6 (13.1)	75.3 (12.1)	75.1 (12.0)
Monitoring days	5.8 (1.6)	5.8 (1.6)	6.0 (1.3)	5.8 (1.5)
Days hospitalized	10.6 (4.6)	10.0 (4.6)	12.6 (5.5)	12.1 (7.6)
Days from stroke to inclusion	0.9 (0.3)	2.4 (0.5)	5.0 (1.3)	2.6 (1.7)
NIHSS when admitted	8.4 (4.4)	8.4 (4.4)	4.6 (6.8)	6.2 (5.0)
Median (IQR)	7.0 (6.0–13.0)	4.0 (3.0–7.0)	3.0 (2.0–5.0)	5.0 (3.0–8.0)
Stroke severity groups, *n* (%)				
Mild (NIHSS < 8)	13 (68.4)	19 (79.2)	14 (93.3)	46 (79.1)
Moderate (NIHSS 8–16)	6 (31.6)	5 (20.8)	0 (0.0)	11 (19.0)
Severe (NIHSS > 16)	0 (0.0)	0 (0.0)	1 (6.7)	1 (1.7)
mRS prior to stroke	1.6 (1.1)	1.6 (1.1)	1.2 (0.9)	1.7 (1.2)
Median (IQR)	1.0 (1.0–2.0)	2.0 (2.0–3.0)	1.0 (1.0–2.0)	2.0 (1.0–2.3)
mRS when admitted	4.1 (0.5)	4.1 (0.5)	4.1 (0.7)	4.1 (0.7)
Median (IQR)	4.0 (4.0–4.0)	4.0 (4.0–5.0)	4.0 (4.0–4.0)	4.0 (4.0–4.3)
Gait speed (m/s)^a^	0.46 (0.40)	0.59 (0.33)	0.58 (0.38)	0.52 (0.36)
SPPB	5.1 (3.8)	5.1 (3.8)	3.9 (3.5)	4.4 (3.5)
Median (IQR)	5.0 (1.0–7.0)	3.5 (2.0–7.0)	4.0 (0.0–7.0)	4.0 (1.0–7.0)

Mean (SD) unless otherwise stated

*NIHSS* National Institute of Health Stroke Scale, *mRS* modified Rankin Scale, *SPPB* Short Physical Performance Battery

^a^These values includes all 58 participants, both those who completed the 4 meter walking test, and those unable to do so (*n*=13). A gait speed of 0 m/s were imputed for participants who not were able to walk 4 meters
